# Elder Abuse During the COVID-19 Pandemic: Prevalence, Risk, and Protective Factors

**DOI:** 10.1093/geroni/igab046.279

**Published:** 2021-12-17

**Authors:** E-Shien Chang, Becca Levy

**Affiliations:** 1 Weill Cornell Medicine, New York City, New York, United States; 2 Yale University, Woodbridge, Connecticut, United States

## Abstract

It has been assumed that the pandemic has brought with it a surge in elder abuse due to heightened health and interpersonal stressors. However, empirical evidence is lacking. This study aimed to estimate the prevalence of, and risk and resilience factors of elder abuse during the pandemic. In a web-based survey of a socio-demographically diverse sample of 897 older persons, one in five older persons (n = 191; 21%) reported elder abuse, an increase of 84% from prevalence estimates before the pandemic. In the multivariate logistic regression models, sense of community was a persistent protective factor for elder abuse (OR= 0.89, 95% CI 0.85–0.93). At the relational level, physical distancing was associated with reduced risk of elder abuse (OR= 0.94, 95% CI 0.90–0.98). At the individual level, financial strain was associated with increased risk of abuse (OR= 1.08, 95% CI: 1.02–1.14). Implications for prevention strategies will be discussed.

